# Image More to Save More

**DOI:** 10.3389/fneur.2015.00156

**Published:** 2015-07-13

**Authors:** Aaron P. Tansy, Jason D. Hinman, Kwan L. Ng, Mateo Calderon-Arnulphi, Royya Modir, Fiona Chatfield, David S. Liebeskind

**Affiliations:** ^1^Department of Neurology, Mount Sinai Comprehensive Stroke Center, New York, NY, USA; ^2^University of California Los Angeles Comprehensive Stroke Center, Los Angeles, CA, USA; ^3^University of California San Diego Comprehensive Stroke Center, San Diego, CA, USA

**Keywords:** CT, MRI, stroke systems of care, acute stroke interventions, endovascular therapy, acute stroke, stroke centers, acute stroke imaging

## Abstract

Recent successful endovascular stroke trials have provided unequivocal support for these therapies in selected patients with large-vessel occlusive acute ischemic stroke. In this piece, we briefly review these trials and their utilization of advanced neuroimaging techniques that played a pivotal role in their success through targeted patient selection. In this context, the unique challenges and opportunity for advancement in current stroke networks’ routine delivery of care created by these trials are discussed and recommendations to change current national stroke system guidelines are proposed.

Recent clinical trials have endorsed a variety of advanced neuroimaging approaches to reiterate the now unequivocal superiority of combined thrombolytic and endovascular therapy for improving outcomes in acute ischemic stroke (AIS) patients with large-vessel occlusion (LVO). Heralding a new era, this momentous advance in treatment has, on the one hand, created a novel challenge to current routine clinical practice and, on the other, a tremendous opportunity to modernize current stroke systems of care: the necessary and inevitable incorporation of advanced imaging techniques into acute stroke. Such integration and utilization, as these trials have demonstrated, holds the key for stroke care providers to save more brain and more stroke patients.

Advanced imaging, specifically vascular imaging, was an essential component of the recent landmark clinical trials and their success. Multicenter Randomized Clinical Trial of Endovascular treatment for AIS in the Netherlands (MR CLEAN), Trial and Cost Effectiveness Evaluation of Intra-arterial Thrombectomy in Acute Ischemic Stroke (THRACE), and Assess the Penumbra System in the Treatment of Acute Stroke (THERAPY) all required imaging evidence of LVO for enrollment (1–3). Even more selectively, THERAPY limited inclusion to LVOs of at least 8 mm in measured length (3). Extending the Time for Thrombolysis in Emergency Neurological Deficits-Intra-Arterial (EXTEND-IA) required not only detection of LVO but also an *a priori* determined favorable perfusion/ischemic mismatch profile within the affected vascular territory (4). Endovascular treatment for small core and proximal occlusion ischemic stroke (ESCAPE) required presence of LVO and excluded those with poor Alberta Stroke Program Early CT Score (ASPECTS) scores and poor collateral circulation (5, 6). Similarly, Solitaire™ FR as primary treatment for acute ischemic stroke (SWIFT-PRIME) and endovascular revascularization with solitaire device versus best medical therapy in anterior circulation stroke within 8 h (REVASCAT) required presence of LVO and excluded those with unfavorable ASPECTS scores (7, 8).

As a consequence of these trials’ requisite inclusion of vascular imaging, their image profiles reflected a more comprehensive, informative assessment of acute stroke than those obtained in routine clinical practice: one not only of tissue status but also of vascular status. More importantly, because these trials enrolled patients with LVO across a wide range of clinical scenarios, their results demonstrated that acute stroke imaging profiles enhanced with vascular status invaluably expanded eligibility for and established treatment of LVO-AIS in its diverse array of clinical impairment beyond what routine practice has offered.

The notion that imaging which reflects both tissue and vascular status may be of great benefit is not new to the field of stroke. An abundance of evidence has progressively mounted to modernize acute stroke management through approaches that provide such information. For one, ASPECTS scoring is a validated method for assessing tissue status using either CT or MR imaging (9) and indicates the likelihood of a favorable response to treatment (5). Vascular status, although less established, has been shown also to play a significant role in AIS (10–12). Collateral flow, in particular, appears to impact acute stroke treatment response: both clinical and radiographic outcomes across all AIS and treatments are better in those with existing collateral flow than in those without (10, 13). As a consequence, the development and utilization of ASPECTS collateral scoring in acute stroke assessment and treatment guidance has been promoted within the stroke community. Furthermore, perfusion-based methods have garnered continued support for assessment of tissue and vascular status in acute stroke (12, 14). Evaluating therapeutic responsiveness for hypoperfusion of an affected territory in LVO, perfusion-based imaging trials have required vascular imaging to determine LVO status for eligibility selection. In fact, EXTEND-IA, where a small ischemic core (<70 cm^3^), a region of hypoperfusion, and a vascular occlusion were required for entry, demonstrated a high-revascularization rate (4) and the lowest NNT (3) of any of the recent trials, supporting the idea that collateral flow and tissue perfusion remain tightly linked to the success of endovascular therapy (15, 16). Even more importantly, ongoing trials utilizing perfusion- and vascular-based imaging have demonstrated promising early results that further encourage and justify continued investigation of imaging profiles in LVO-AIS that may be most responsive to recanalization therapies (17).

In essence then, advanced stroke imaging has changed how providers can now utilize diagnostic methods to inform treatment decision-making, whereas before it allowed for exclusion of pathology (i.e., hemorrhage) (18), it now allows for active detection of it (i.e., LVO, ischemic changes). This revolution in applicability affords, somewhat paradoxically, the opportunity to deliver more and better care, but only at the expense of improved diagnostic certainty not obtained in routine clinical practice. As a consequence, the modernization of acute stroke through utilization of advancing neuroimaging requires a re-evaluation of acute stroke triage and available diagnostic resources within the hub-and-spoke model.

Current stroke systems of care predominantly implement a hub-and-spoke model that links multiple primary stroke centers (PSCs) with a comprehensive stroke center (CSC) (19). This model provides proven excellence in stroke care for uncomplicated cases at all sites through compliance with established best-care practice, but also allows for a higher level of care for more complicated cases at CSCs when necessary (20). Best-care practice required for PSC designation includes immediate neuroimaging availability for determination of thrombolysis eligibility, largely achieved with non-contrast CT. However, more advanced neuroimaging approaches, such as multimodal CT or MRI to ascertain vascular and perfusion status, are presently not required.

Consequently, these requirements already provide challenge to current consensus positions on early management of LVO-AIS. The 2013 AHA/ASA Guidelines for the Early Management of Patients with AIS include the following recommendations: intracranial vascular imaging when endovascular therapy is considered (Class I, LOE A) and perfusion-based methods for reperfusion therapies when event duration exceeds thrombolytic eligibility windows (Class IIb, LOE B) (19). This challenge is only magnified by the fact that the vast majority of patients receive their initial acute stroke evaluation at PSCs: according to the “Get with the Guidelines” registry data from 2014, over 70%. Furthermore, although LVO comprises only a minority of this population, it carries the highest rates of disability making its rapid identification and treatment crucial (21). As a consequence, efficient triage and selection of LVO-AIS for potential combined or endovascular monotherapy cannot rely on nor succeed with the existing imaging standards of PSCs. Because advanced imaging has now become a key determinant in stroke treatment best-practice, incorporation of such methods, particularly vascular imaging, and their rapid expert interpretation have become a necessity of all designated stroke centers.

Without updating this requirement for PSC designation, the current framework within which stroke care is delivered faces significant challenges. For one, currently designated PSCs without at least vascular imaging capability and vascular neurology expertise available for its interpretation run the grave risk of becoming obsolete. Although these sites can administer thrombolytic therapy and clinically infer presence of LVO, without vascular imaging and its expert evaluation, they can no longer provide a definitive, complete assessment of acute stroke rendering them ineffective within an acute stroke system of care. In fact, a recent analysis of over 11,000 patients in the SITS-International Stroke Thrombolysis Register demonstrated that an NIHSS of 11 was moderately predictive of LVO, though the sensitivity of this measure was only 64.5% (22), in line with prior studies suggesting that this widely used and PSC-certification-required initial triage assessment tool is not adequate to identify all patients with LVO (23). Thus, this handicap will have many downstream effects within acute stroke networks diminishing stroke care delivery overall: *a priori* bypassing of centers without access to vascular imaging and/or additional transfer to those with it leading to the disuse of certain centers and an overburdening on and stressing of a network’s remaining available sites, services, and resources to accommodate this need.

With these concepts in mind, we suggest that PSC certification (or re-certification) mandate the following new key elements: (1) immediate availability of vascular imaging with either contrast-enhanced CT angiography or time of flight magnetic resonance angiography for all patients presenting with acute stroke; (2) immediate availability of vascular neurology expertise via in-person or telemedicine for clinical and radiologic evaluation of acute stroke; and (3) in-place protocols within acute stroke networks of care for rapid identification, stabilization, and transfer of LVO-AIS patients to CSCs or facilities of equivalence in care.

The colossal efforts to advance acute stroke care have yielded a tremendous opportunity that should not be forsaken. More imaging, incorporating non-invasive angiography and multimodal CT or MRI, beyond the current standard of non-contrast CT at PSCs will facilitate triage of stroke patients for current state-of-the-art therapies to save more brain and to extend this opportunity to more patients at greatest risk of long-term disability. Such modernization of stroke systems of care through incorporation of advanced imaging methods and their timely interpretation in clinical context is not just an opportunity, but an inevitable next step that recent trial success has galvanized with a clear message: we must image more to save more.

## Author Contributions

AT – conceived of, principally wrote, and revised the manuscript. JH – conceived of, principally wrote, and revised the manuscript. KN – intellectual contribution through drafting or revising the manuscript for intellectual content. MC-A – intellectual contribution through drafting or revising the manuscript for intellectual content. RM – intellectual contribution through drafting or revising the manuscript for intellectual content. FC – analyzed data. DL – conceived of, principally wrote, and revised the manuscript.

## Conflict of Interest Statement

Dr. Aaron P. Tansy reports the following disclosures: He serves as an editorial board member of *Neurocritical and Neurohospitalist Care*, a section of the journal Frontiers in Neurology. Dr. Jason D. Hinman reports the following disclosures: He serves as an editorial board member of *Journal of Neurological Disorders* and *Neurocritical and Neurohospitalist Care*, a section of the journal Frontiers in Neurology; and is supported by NIH grant #NS083740. Dr. Kwan L. Ng reports the following disclosures: He is supported by the American Heart Association grant #14BFSC17760005 ASA-Bugher Stroke Center. Dr. Mateo Calderon-Arnulphi, Dr. Royya Modir and Ms. Fiona Chatfield report no disclosures. Dr. David S. Liebeskind reports the following disclosures: He is a paid consultant for Stryker and Covidien, and is supported by NIH grant #K24NS072272.
